# Tick-borne encephalitis infections without CNS involvement: An observational study in Latvia, 2007–2022

**DOI:** 10.1371/journal.pone.0305120

**Published:** 2024-06-07

**Authors:** Zane Freimane, Guntis Karelis, Maksims Zolovs, Dace Zavadska

**Affiliations:** 1 Department of Paediatrics, Children’s Clinical University Hospital, Riga Stradiņš University, Riga, Latvia; 2 Department of Neurology and Neurosurgery, Riga East University Hospital, Riga, Latvia; 3 Department of Infectology, Riga Stradiņš University, Riga, Latvia; 4 Statistics Unit, Riga Stradiņš University, Riga, Latvia; 5 Institute of Life Sciences and Technology, Daugavpils University, Daugavpils, Latvia; DIU: Dhaka International University, BANGLADESH

## Abstract

**Background:**

Tick-borne encephalitis (TBE) is a human viral infectious disease involving the central nervous system (CNS). It is caused by the tick-borne encephalitis virus (TBEV). At present, there is very limited information regarding the clinical importance and health burden of TBE infections without signs of CNS inflammation. Moreover, such cases are omitted from official TBE surveillances and there are no reports of population-based studies.

**Methods and findings:**

A nationwide population-based study was conducted in Latvia by intensively searching for symptomatic TBEV infections recorded in outpatient and hospital settings between 2007 and 2022. In total, 4,124 symptomatic TBEV infections were identified, of which 823 (20.0%) had no CNS involvement. Despite the lack of neurological symptoms, non-CNS TBE patients still experienced severe health conditions that required management in a hospital setting for a median duration of 7 days. Furthermore, lumbar puncture information was available for 708 of these patients, with 100 (14.1%) undergoing the procedure, suggesting a high suspicion of CNS involvement.

**Conclusions:**

Clearly, non-CNS TBE has the potential to negatively impact the health of patients. The actual burden of non-CNS TBEV cases may be higher than we think as these cases are omitted from official TBE surveillances and are challenging to recognize.

## Introduction

Tick-borne encephalitis (TBE) is a human viral infectious disease involving the central nervous system (CNS). It is caused by the tick-borne encephalitis virus (TBEV). Infected ticks usually transmit TBEV via tick bites or, in rare circumstances, by consumption of unpasteurized dairy products from infected goats, sheep or cows. TBE is endemic in northern, central and eastern Europe and Asia [1]. For the year 2020, 24 European countries reported 3,817 cases of TBE, of which 3,734 (97.8%) were confirmed [2]. Latvia has one of the highest annual TBE incidences in Europe– 12.6/100,000 population per year (2018–2020) [3]. Although the disease is preventable by vaccination, it continues to be one of the most frequent causes of viral meningitis/encephalitis in endemic countries among visitors and the local population [4, 5].

TBE is usually a biphasic disease manifesting with influenza-like febrile illness during the first viremic phase, followed by the second viremic phase with neurological symptoms of different severity, ranging from meningitis to severe neurological forms like encephalitis and myelitis [6]. According to the published data, 5–50% of symptomatic TBEV infections manifest without CNS involvement [7]. In the literature, these non-CNS cases of TBE have been termed “fever form”, “abortive form”, “isolated initial phase of TBE” or “febrile illness” and are defined by the presence of fever and constitutional symptoms and the absence of clinical signs of CNS involvement at the time of illness [8]. A recent prospective follow-up study from Slovenia characterized patients with TBEV infection after a tick bite or tick exposure. Only 4 of 62 patients (6%) experienced mild symptoms not associated with meningeal signs and 6 (10%) remained asymptomatic [8]. These findings do not appear to support the notion that TBE without CNS involvement is a frequent clinical manifestation of an infection with TBEV. However, possible selection bias may have occurred as patients with more severe disease or unusual laboratory findings were more likely referred to the study.

In Europe, the non-CNS TBE form is not well recognized due to the European Centre for Disease Prevention and Control’s (ECDC) official case definition, i.e. it is not mandatory for TBE without signs of CNS inflammation to be reported and included in official surveillances. Consequently, published data show that the health burden of non-CNS TBE cases might be underestimated. For instance, a study from Latvia reported that non-CNS TBE cases accounted for 27.1% of all serologically confirmed TBEV infections in the country between 2007 and 2016 [9]. Furthermore, a recent study from Sweden showed that patients with a TBEV infection history (including non-CNS cases) were hospitalized for significantly longer (11.5 versus 1.1 days) during the first year after disease onset compared to the control cohort with no TBE history. Additionally, more specialist outpatient visits (3.6 versus 1.2 visits) and more sick leave days (66.0 versus 10.7 days) were logged for TBEV infections [10]. The authors of the study concluded that the health impact and healthcare costs of TBEV infections are far greater than estimated.

At present, there is no clear consensus regarding the clinical importance and health burden of TBE infections without signs of CNS inflammation. The literature provides very little information on the non-CNS TBE form and there are no reports of population-based studies. Here, we analysed population-level data on all reported non-CNS TBE forms from the largest TBE surveillance study in Latvia covering the time period 2007 to 2022. We assessed the epidemiological, clinical and laboratory characteristics of TBE infections without CNS involvement and subsequently determined the clinical importance and short-term health risk they represent.

## Subjects and methods

### Study design and population

We conducted a cross-sectional descriptive study by intensively searching for all symptomatic TBEV infections recorded in Latvia between 2007 and 2022, specifically identifying those without CNS inflammation signs and symptoms. Data from a retrospective nationwide TBE surveillance study (2007–2017) in Latvia were supplemented with data from an ongoing prospective nationwide TBE surveillance study (2018–2022) involving the same institutions. The study population included all persons with a laboratory-identified symptomatic TBEV infection who sought medical attention in Latvia (overall country population of 1.85–2.20 million during the study period).

### Data collection

For the retrospective data collection covering the years 2007 to 2017, the Centre for Disease Prevention and Control (CDPC) of Latvia provided all the TBEV infection cases in their electronic database to our study investigators at Riga Stradiņš University. National legislation ensures that a countrywide mandatory case-based passive reporting system exists in Latvia, presided over by the CDPC of Latvia. Hospitals across Latvia were contacted and asked to provide the respective patients’ case histories/medical records for additional clinical data. In total, 26/27 Latvian hospitals provided TBE case medical records for chart review; one hospital refused to share their TBE case medical records. Further data collection from patient records was guided by a questionnaire that included information about age, gender, region of residence, TBE vaccination history (including the number of doses received and the vaccination date of the most recent dose), tick bite history, exposure to the same identified infected food source as a confirmed TBE case during an outbreak, clinical symptoms, laboratory test results, lumbar puncture results, duration of hospitalization and clinical outcome at discharge (TBE form). Retrospective data collection for 2007–2016 TBE cases was carried out between November 1^st^, 2016, and March 31^st^, 2017 (Ethics Committee Approval No. 24 / 08.09.2016). Retrospective data collection for 2017 TBE cases was carried out between June 1^st^, 2018, and August 15^th^, 2018 (Ethics Committee Approval No. 21 / 29.03.2018). Only data from the CDPC electronic database were accessed.

For the prospective data collection covering the years 2018 to 2022, an active, intensive, nationwide search for any symptomatic TBEV infection in Latvia was conducted, starting from January 1^st^, 2018, until December 31^st^, 2022 (Ethics Committee Approval Nos. 2 / 30.11.2017 and 22 / 2/27/2021). The CDPC and Latvia’s National Microbiology Reference Laboratory informed Riga Stradiņš University study investigators of individuals with laboratory-identified TBEV infections. In addition, study investigators conducted active surveillance for hospitalized TBEV-infected persons at the 15 largest hospitals in the country. Due to reform of the Latvian hospital network in 2020, there were 15 hospitals, where a patient with a suspected TBEV infection was most likely to be hospitalized during the period of prospective study. After informed consent from a TBEV-infected person, study investigators collected information on age, gender, region of residence, risk factors, recent tick bite or consumption of unpasteurized dairy products, occupation, hospitalization and TBE vaccination history (including vaccine product and administration date) via interviews, medical record review and review of vaccination cards. Hospitalized TBEV-infected persons were followed to determine a detailed clinical picture, laboratory test results, duration of hospitalization, intensive care unit (ICU) admission, clinical outcome and discharge status. For patients that died, the recorded cause of death was collected.

### Statistical analysis

From all reported TBEV infections between 2007 and 2022 in Latvia, non-CNS TBE cases were analysed further. TBE cases with CNS symptoms were excluded from the main analysis. However, some data were analysed for comparison purposes between CNS (meningitis etc.) and non-CNS cases. To describe the data (annual incidence, age group, gender, immunization status, region of Latvia, clinical outcome of the cases, etc.), descriptive statistics were calculated: 1) frequency, 2) percentage, 3) centre point of the data (median) and 4) spread of the data (interquartile range (IQR, presented as the first and third quartiles) and min–max values).

The inferential statistics were calculated to test statistical hypotheses. The chi-squared test was used to test the proportion of non-CNS cases between regions and seasons. The Mann-Whitney U test was used to compare seasonal patterns between non-CNS and CNS TBE cases.

Subjects of any age were included and categorized into two main groups: children (<18 years) and adults (≥18 years). More detailed clinical data analyses were performed in three age groups (<18 years; 18–65 years; >65 years). Statistical analysis of the data was conducted using the jamovi statistical platform [11].

### Definitions

*TBE case*–defined according to the ECDC Case Definition Criteria: a patient with symptoms of inflammation of the CNS (meningitis, meningoencephalitis, etc.) and demonstrating TBEV-specific IgM antibodies or both TBEV-specific IgM and IgG antibodies [12].

*TBE infection*–a patient with any clinical symptoms demonstrating TBEV-specific IgM antibodies or both TBEV-specific IgM and IgG antibodies.

*Non-CNS TBE case*–a patient without any clinical symptoms of CNS inflammation and demonstrating TBEV-specific IgM antibodies or both TBEV-specific IgM and IgG antibodies.

### Ethical conduct of the study

The study was conducted in full compliance with legal and regulatory requirements. Ethical approval was determined by the Riga Stradiņš University Ethical Commission: for retrospective data collection–No. 24 / 08.09.2016, No. 21 / 29.03.2018; for prospective data collection–No. 2 / 30.11.2017, No. 22 / 2/27/2021.

Written consent was obtained from adults and the parent/legal guardian of children prior to data collection for the prospective part of the study (covering the years 2018–2022). Retrospective data collection (covering the years 2007–2017) was a non-interventional, observational process consisting of secondary analysis of anonymized data; thus, informed consent was not required.

## Results

### Epidemiological data

**Fig 1:**
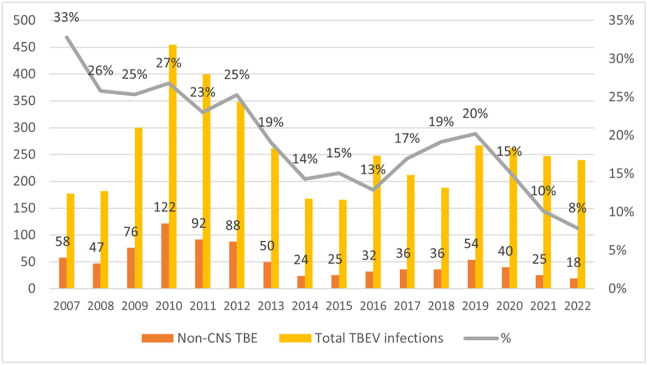
Non-CNS TBE cases and total TBEV infections in Latvia, 2007–2022; N, %.

In total, 4,124 TBE infections were identified in Latvia between 2007 and 2022 (Latvian population: 1.85 million in 2022) [13]. The annual incidence of TBE cases was 10.6/100,000 population, ranging from 5.9/100,000 (2007) to 14.6/100,000 (2010). Of these 4,124 reported TBEV infections, 823 were non-CNS TBE cases who experienced symptoms not associated with meningeal signs. In 100 cases, CNS inflammation signs were ruled out by a lumbar puncture–negative leukocyte count <5 × 10^6^/L and negative TBEV-specific IgM antibodies in cerebrospinal fluid. Non-CNS TBE cases represented 20% of all serologically confirmed TBEV infections during this period (Fig 1).

### Regional distribution

Non-CNS TBE cases were reported from all regions of Latvia during the time period 2007 to 2022. The southern and southeastern parts of Latvia had the highest proportions of non-CNS cases among all reported TBEV infections; Zemgale 100/408 (24.5%) and Latgale 65/301 (21.6%). Slightly lower proportions were found in the capital Riga/Central area (246/1,207 (20.4%)); Kurzeme 247/1,310 (18.9%) and Vidzeme 154/800 (19.3%). No significant differences were found among the different regions of Latvia (p>0.05).

In addition, the proportions of non-CNS TBE cases were compared between the western and eastern parts of Latvia (left and right banks of the Daugava River), where different TBEV subtypes might be circulating. However, no significant difference was detected; western part (Kurzeme+Zemgale+Riga regions) 593/2,925 (20.3%) and eastern part (Vidzeme+Latgale) 219/1,101 (19.9%) (p>0.05).

### Seasonality

Data concerning the onset of non-CNS TBE countrywide by month were available for 96.3% (n = 793) of patients. A statistically significant difference was found between the seasonal patterns of non-CNS infections (X^2^(11) = 862.8, p<0.001). Most non-CNS cases were reported during the summer months– 465/793 (58.6%); June 130 cases (16.4%); July 145 cases (18.3%), August 190 cases (24.0%). In autumn, 247 non-CNS cases (31.1%) were reported, with 153 cases (19.3%) in September alone. During spring, 76 cases (9.6%) were reported, while only 5 (0.6%) were reported in winter. However, the seasonal patterns were not significantly different between non-CNS and CNS TBE cases (p>0.05).

### Risk factors

A tick bite was noticed in 62.5% of non-CNS TBE cases (499/798) and 59.5% of CNS TBE cases (1,855/3,120) (p>0.05). Transmission of TBEV via unpasteurized cow and goat dairy products was detected in 1.1% of non-CNS TBE cases (9/805). Occupational and leisure activity risk factor information was available for 172/173 non-CNS TBE cases for the time period 2018 to 2022. Occupational risk factors were detected in 10.6% of cases (16/151): forest worker (7 cases), farmer (7 cases) and road construction worker (2 cases). Leisure activity risk factors were noted in 172/173 non-CNS cases; 80.8% of cases (139/172) had outdoor activities in the country/nature and 38.4% of cases (66/172) mentioned outdoor activities in the city.

### Patient characteristics

The median age of the non-CNS TBE cases was 51 years (Q1–Q3 35 to 63 years); 53 of the cases (6.4%) were children. Of all reported TBEV infections in children, non-CNS TBE accounted for 20.9% (53/254). In adults, non-CNS TBE accounted for 19.9% (770/3,868); specifically, 19.4% (586/3,026) in the 18–65 years age group and 21.9% (184/842) in the >65 years age group. No significant differences were found among the age groups (p>0.05).

Interestingly, non-CNS cases were more frequently seen in females (51.3%, 422 cases) than in males (48.7%, 401 cases). In contrast, CNS cases were more frequent in males than in females (53% versus 47%).

Information regarding underlying health conditions was available for 173 non-CNS TBE patients for the time period 2018 to 2022. An underlying chronic health condition was reported for 40 patients; of these patients, 17 (42.5%) had more than one chronic disease. The most common chronic health conditions were cervical spondylosis (15 cases), arterial hypertension (12 cases), chronic heart disease (4 cases), diabetes mellitus (3 cases), epilepsy, cognitive disorder and migraine.

### Vaccination status

Vaccination status was known for 820 of the 823 non-CNS TBE patients (99.6%). Of these 820 patients, 19 (2.3%) were vaccinated with at least one prior dose and 801 (97.7%) were not vaccinated. Previously vaccinated subjects were more common among non-CNS TBE cases than CNS cases, where 1.7% (54/3,215) were found to be vaccinated (p = 0.204). Non-CNS TBE cases with a prior vaccination history received a median of three doses and disease onset four years after the last dose. According to the received dose schedules of the 19 non-CNS cases, 6 (31.6%) were vaccinated according to the recommended schedule (vaccine failure), 11 (57.9%) were improperly vaccinated due to non-adherence to the recommended schedule (vaccine ineffectiveness) and 2 had a missing schedule.

### Clinical presentation

Detailed clinical information was available for the prospective part of the study (2018–2022). During this time period, a total of 1,204 TBEV infections were reported in Latvia, of which 173 (14.4%) were non-CNS TBE cases. In total, 88 (50.9%) non-CNS TBE patients consented to a clinical examination. The median incubation period was 17 days (range 3 to 58 days). The most common general symptoms were fatigue 90.9% (n = 80), fever ≥38°C 89.8% (n = 79), headache 84.1% (n = 74), dizziness 56.8% (n = 50), nausea/vomiting 44.3% (n = 39) and myalgia 36.4% (n = 32) (Table 1). Additionally, two patients had nuchal rigidity symptoms from an underlying health condition (cervical spondylosis) that mimicked meningeal signs; however, they were not categorized as CNS cases.

**Table 1:** Demographic and clinical characteristics of non-CNS and CNS TBE cases in Latvia, 2018–2022; N = 1,204*.

Characteristics	Non-CNS TBE cases (N = 173)	CNS TBE cases (N = 1,031)
*Patient characteristics*		
Median age, y (IQR)	Median = 51 (Q1–Q3 35–63)	Median = 51 (Q1–Q3 35–63)
Sex: Female	92/173 (53.2%)	453/1,031 (43.9%)
Male	81/173 (46.8%)	578/1,031 (56.1%)
Underlying illnesses	40/173 (23.1%)	156/877 (17.8%)
Vaccination history	5/173 (2.9%)	16/1,031 (1.6%)
Tick bite noticed	95/173 (54.9%)	580/1,031 (56.3%)
Consumption of unpasteurized dairy products	1/172 (0.6%)	1/1,029 (0.1%)
Occupational risk factors	16/151 (10.6%)	171/1,009 (16.9%)
*TBE general symptoms*		
Median incubation period, d	Median = 17	Median = 17
Headache	74/88 (84.1%)	656/687 (95.5%)
Fever	79/88 (89.8%)	678/687 (98.7%)
Myalgia	32/88 (36.4%)	283/687 (41.2%)
Arthralgia	24/88 (27.3%)	191/687 (27.8%)
Nausea/vomiting	39/88 (44.3%)	412/687 (59.9%)
General fatigue	80/88 (90.9%)	663/687 (96.5%)
Photophobia	24/88 (27.3%)	236/687 (34.4%)
Phonophobia	10/88 (11.4%)	124/687 (18.0%)
Dizziness	50/88 (56.8%)	439/687 (63.9%)
Erythema	0/88 (0%)	3/687 (0.4%)
*Hospitalization*		
Hospitalized	159/173 (91.9%)	1,031/1,031 (100%)
Median hospitalization length, d (IQR)	Median = 7 (Q1–Q3 4–9.25)	Median = 10 (Q1–Q3 8–13)
Initial clinical diagnosis–TBE	129/169 (76.3%)	464/1,030 (45.0%)
ICU admission	0	23/835 (2.8%)
*Outcome*		
Subject recovered	159/160 (99.4%)	937/1,008 (93.0%)
Death	0	15/1,031 (1.5%)

* TBEV infections with unknown demographic and clinical data were excluded from the analysis. Detailed clinical information was available only for those patients (2018–2022) who gave informed consent and underwent neurological examinations.

A total of 159 (91.9%) of the 173 non-CNS TBE cases reported between 2018 and 2022 were hospitalized. The median hospitalization length was 7 days (Q1–Q3 4 to 9.25 days), with a range of 1 to 43 days (Table 1). None of the non-CNS cases was admitted to the ICU.

TBE as the initial clinical diagnosis was considered in 129/169 cases (76.3%). In 4 cases, information about an initial clinical diagnosis was missing. Other common differential diagnosis factors included unspecified fever (Diagnosis Code R50.9, 13 cases), unspecified viral infection (Diagnosis Code B34.9, 4 cases), pneumonia (4 cases) and acute upper respiratory tract infection (4 cases) (Table 2).

**Table 2:** Initial clinical diagnoses for non-CNS TBE cases, 2018–2022; N = 169.

*Initial clinical diagnoses*	*Number of cases*, *%*
TBE	129 (76.3%)
Fever	13 (7.7%)
Viral infection	4 (2.4%)
Pneumonia	4 (2.4%)
Acute upper respiratory tract infection	4 (2.4%)
Urinary tract infection	3 (1.8%)
Infectious gastroenteritis and colitis	2 (1.2%)
Epilepsy	2 (1.2%)
Influenza	1 (0.6%)
Migraine	1 (0.6%)
Viral meningitis	1 (0.6%)

### Laboratory tests

Detailed serological test results were available for 88 non-CNS TBE patients from the prospective part of the study (2018–2022). Analysis of initial serum samples revealed both positive TBEV-specific IgM and IgG antibodies in 71 (80.7%) patients and only positive TBEV-specific IgM antibodies in 17 (19.3%) patients.

Lumbar puncture information was available for 708 non-CNS TBE patients (2007–2022), with 100 (14.1%) undergoing the procedure. Specifically, between 2018 and 2022 (the prospective part of the study), 19 out of 88 patients (21.6%) underwent a lumbar puncture. Cerebrospinal fluid pleocytosis (>5 × 10^6^ leukocytes/L) was negative in all these cases. A control lumbar puncture was performed in four non-CNS cases; negative pleocytosis in the cerebrospinal fluid was confirmed.

## Discussion

TBE is an emerging public health issue in Latvia, with an annual incidence of 10.6 per 100,000 population (1.9 million overall population) or approximately 200 new TBE cases yearly. However, as only CNS cases (meningitis, encephalitis, etc.) are included in the official nationwide TBE surveillance, this number of TBEV-infected persons is potentially much greater and at present not a realistic estimate. According to the official case definition adopted in Europe since 2012, the reporting of symptomatic TBE infections without signs of CNS inflammation is not mandatory. In order to more fully understand the clinical importance and health burden of non-CNS TBE cases, we conducted a nationwide population-based study in Latvia covering the time period 2007 to 2022 by intensively searching for symptomatic TBEV infections recorded in outpatient and hospital settings. In total, 823 symptomatic TBEV infections without CNS involvement were identified, representing one-fifth (20%) of all TBEV infections in the country. Our data confirm that the TBE burden on public health is indeed higher than estimated by the official nationwide TBE surveillance data. Moreover, the true denominator/number of all infected individuals might be even higher as TBEV infections without CNS involvement are more challenging to recognize [14].

The reported TBE infections without CNS involvement manifested as a clinical array of various non-specific symptoms, mostly general fatigue 90.9%, fever 89.8%, headache 84.1%, dizziness 56.8%, nausea/vomiting 44.3% and myalgia 36.4%. Two patients also presented with nuchal rigidity; however, meningeal syndrome and CNS involvement were ruled out after a detailed medical history and clinical/laboratory data evaluation. Despite the lack of neurological symptoms, patients still experienced conditions that required management in a hospital setting for several days; 92% of non-CNS cases were hospitalized, on average, for 7 days. Furthermore, during the time period 2018 to 2022, one-fifth of patients (21.6%) underwent a lumbar puncture; it was one-seventh (14.1%) between 2007 and 2022. This suggests a higher suspicion of CNS inflammation in patients with fever, headache and other general symptoms.

Interestingly, our data have also revealed a tendency towards a lower proportion of non-CNS cases among all reported TBEV infections in Latvia in recent years. Specifically, over a 16-year period, the proportion fell from 33% in 2007 to only 8% in 2022. This may be due to increased TBE awareness in the country and/or improved diagnostic efforts to distinguish between CNS and non-CNS clinical forms (e.g. lumbar puncture). Further investigations need to be conducted.

Analysis of initial serum samples demonstrated that 80.7% of non-CNS TBE patients already had both positive TBEV-specific IgM and IgG antibodies and 19.3% had only positive TBEV-specific IgM antibodies. In the majority of patients, antibody production suggests a second viremic phase of TBE and that non-specific influenza-like symptoms cannot always be assumed as prodromal or the first viremic phase. Possible organ damage should be considered and diagnostic methods applied covering both phases, the primary one being the detection of TBEV-specific antibodies in any symptomatic TBEV infection. The role of a lumbar puncture in TBEV infections without apparent CNS symptoms is debatable, i.e. whether positive pleocytosis and positive TBEV-specific IgM antibodies in cerebrospinal fluid should be excluded. More data are necessary to fully understand the complexity of CNS inflammation symptom recognition in TBE patients and situations when CNS inflammation diagnosis is based solely on lumbar puncture results.

TBE almost entirely occurred in unvaccinated individuals. For our time period of study (2007–2022), 97.7% of non-CNS patients were unvaccinated against TBE. Although vaccine failure was a rare phenomenon, the proportion of vaccinated individuals was higher among non-CNS cases than CNS cases (2.3% versus 1.7%). This may indicate that if vaccination does not prevent TBE completely in an individual, it may lower the individual’s disease severity. Indeed, a recent study on TBE vaccine failure in Latvia (2007–2019) demonstrated that mild cases (non-CNS TBE) were relatively more frequent among TBE-vaccinated cases (39%) than non-TBE-vaccinated cases (25%) [15]. TBE vaccination has proven highly effective in a recent nationwide population-based study conducted in Latvia between 2018 and 2020. Specifically, vaccine effectiveness was estimated at >99% in preventing medically attended TBEV outcomes, including hospitalization and infection with non-CNS symptoms [16].

This study has some limitations. We used a self-adapted case definition for non-CNS TBE cases as such cases have not previously been defined in the literature. Our criteria included (1) serological identification of TBEV infection by demonstration of TBEV-specific antibodies and (2) lack of CNS inflammation symptoms. However, we know that meningeal symptoms might be clinically indistinguishable in some patients and lumbar puncture is the most accurate way to exclude CNS inflammation evidence. Nevertheless, study investigators obtained a detailed medical history whenever possible and only observed the disease management process according to the study design. Another limitation is that the number of non-CNS TBE cases in the outpatient setting might be higher as TBE is not always considered nor tested for when an individual presents with a febrile illness.

## Conclusions

This study shows that non-CNS TBE disease can potentially negatively impact a patient’s health. In 92% of all recognized non-CNS TBE infections in Latvia between 2018 and 2022, patients experienced serious illness that required management in a hospital setting for an average of 7 days. Furthermore, in 14.1% of non-CNS TBE cases (2007–2022), lumbar puncture was performed, suggesting a high suspicion of CNS involvement. Moreover, the true health burden of non-CNS TBE cases might be even higher as these cases are omitted from official TBE surveillances and clinically milder forms of the disease are challenging to recognize.

In order to improve non-CNS form recognition and true health estimates in TBE endemic countries, we suggest the implementation of a high suspicion of TBEV infection for all patients who meet the following criteria: (1) present during tick activity season; (2) present with a tick bite history; (3) present with clinically non-specific symptoms, especially fever, headache, general fatigue, dizziness, nausea/vomiting and myalgia; (4) not vaccinated against TBEV. Additionally, there needs to be advancement in the facilitation and promotion of science communication across the different TBE endemic regions to greatly improve knowledge of the disease.
